# Femtosecond Laser Plane-by-Plane Inscribed Cavity Mirrors for Monolithic Fiber Lasers in Thulium-Doped Fiber

**DOI:** 10.3390/s21061928

**Published:** 2021-03-10

**Authors:** Antreas Theodosiou, Jan Aubrecht, Ivan Kašík, Daniel Dousek, Matěj Komanec, Kyriacos Kalli

**Affiliations:** 1 Lumoscribe LTD, Margaritas Liasidou 12, Paphos 8310, Cyprus; 2 Photonics and Optical Sensors Research Laboratory (PhOSLab), Cyprus University of Technology, Saripolou 33, Limassol 3036, Cyprus; kyriacos.kalli@cut.ac.cy; 3 Institute of Photonics and Electronics of the Czech Academy of Sciences, Chaberská 57, 182 51 Prague, Czech Republic; aubrecht@ufe.cz (J.A.); kasik@ufe.cz (I.K.); 4 Department of Electromagnetic Field, Czech Technical University in Prague, Technicka 1902/2, 166 27 Prague, Czech Republic; dousedan@fel.cvut.cz (D.D.); komanmat@fel.cvut.cz (M.K.)

**Keywords:** fiber Bragg gratings, monolithic fiber lasers, femtosecond laser, fiber lasers

## Abstract

A monolithic fiber laser operating in the short wavelength infrared that is suitable for CO_2_ gas sensing applications is proposed and presented. The current study reports a laser design based on the direct inscription of a monolithic Fabry–Perot (FP) cavity in a thulium-doped optical fiber using the femtosecond laser (FsL) plane-by-plane inscription method to produce the cavity mirrors. The FP cavity was inscribed directly into the active fiber using two wavelength-identical fiber Bragg gratings (FBGs), one with high and one with low reflectivity. Initially the effective length of the fiber was defined using a single high reflectivity FBG and subsequently a very weak FBG was inscribed at the other end of the fiber in order to demonstrate a fully monolithic fiber laser. All fiber lasers were designed for continuous wave operation at 1950 nm and characterized with respect to the power output, slope efficiency, stability, and effective resonator length. The performance of the presented monolithic laser cavities was evaluated using the same active fiber as a reference fiber spliced to FBGs inscribed in passive fiber; an improvement exceeding 12% slope efficiency is reported for the presented monolithic laser.

## 1. Introduction

Lasers are highly coherent monochromatic light sources that have impacted our lives in a myriad of ways for more than 50 years. Lasers are ubiquitous and have proved to be essential tools for many fields and applications. Key laser-based technologies are in communication and data storage systems, medical applications, and related diagnosis, treatment, and surgical procedures [1,2]. Lasers are critical tools for applications in meteorology, spectroscopy, and microscopy measurements [3].

Lasers consist of three principal parts, the active gain medium, the wavelength selective reflection mirrors, and pump light source. The light source at a specific wavelength excites atoms of the active medium. The operating wavelength of the laser is defined by the material’s energy bands of the active gain medium. In recent years, fiber lasers (FL) have been strongly promoted, as the optical fibers can be readily doped with controlled quantities of rare earth elements such as erbium, ytterbium, neodymium, thulium, and holmium [4,5]. The benefits of using the optical fiber as the gain medium are various, among them the efficient cooling due to the extended length of the laser cavity, compact size, high reliability for a high output power, stability, and, importantly, the light is fundamentally coupled within a flexible fiber for easy and secure light delivery.

A critical component to realize the aforementioned advantages of FL is the use of fiber Bragg gratings (FBGs) to define the laser cavity, in contrast to conventional dielectric mirrors [6,7,8]. The FBGs are excellent laser reflectors and their optical characteristics, such as reflectivity, resonance wavelength, and bandwidth, can be tailored appropriately to the requirements of the laser system. The FBG technology is mature and has been used for sensing and other applications for many years. Different grating inscription techniques have been developed, including interferometric and phase mask inscription, principally employing continuous wave ultraviolet lasers [9,10,11] and point-by-point inscription methods that use femtosecond lasers (FsL) [12,13]. FBGs can be readily inscribed with reflectivities exceeding 99.99% and a bandwidth less than 0.4 nm in common, single mode silica fibers.

In typical FL configurations, the FBG is inscribed in silica fibers and carefully spliced to the active fiber. However, most of the FL failures are observed at the spicing joints that are very susceptible to damage as a result of high-power fluctuations and thermal effects. Hence, the monolithic inscription of FBGs directly in the active gain fiber is very important in order to minimize these failures, whilst also leading to the development of more powerful laser systems. The monolithic inscription of the Bragg components are mostly performed using high intensity femtosecond laser pulses, for the periodic modification of the fiber core refractive index (through the multiphoton absorption), without the need of stripping and recoating the fiber buffer coating.

However, the high-pulse-energy FsL inscription methods, such as point-by-point (PbP) [14,15,16,17] and phase mask methods [18,19,20], introduce high attenuation and polarization losses leading to poor laser efficiencies. The high alignment requirements of the PbP method result in repeatability issues and wavelength mismatching between the two FBGs reflectors. On the other hand, the FBG produced with a phase mask is largely limited in wavelength to the predefined period of the phase mask.

Thulium fiber lasers (Tm-FL) operating with laser wavelengths between 1.6 to 2.1 μm [4,21,22,23] were proposed for various applications, including as a biomedical tool for tissue soldering [24] and lithotripsy [25], while they are considered as highly efficient lasers for material processing applications, especially for polymer processing [26,27] since the emission bands of Tm-lasers coincide with the absorption bands of the polymers [28]. In addition, their applications are extended to greenhouse atmospheric gas sensing [29,30], such as the measurement of CO_2_ concentration. It is well known that CO_2_ has a strong fingerprint between 1.8 μm and ~2 μm [31], which coincides with the emission band of Tm-lasers. As a result, the efficient monolithic inscription of the FBGs is very important for more robust and cost-effective fiber laser systems. In particular, for gas sensing applications the good directionality and beam quality of the fiber lasers in combination with the compact design make them ideal when it comes to the real-world application measurements.

In theory, the Tm-FL takes advantage of the cross-relaxation process when pumped at 792 nm, and can achieve slope efficiencies up to 80% [32]. However, in practice the highest efficiency reported was 68% using dichroic thin films as reflectors on the end-face of optical fibers in 2007 [33] and up to 71% in 2009 using a master-oscillator power-amplifier (MOPA) design [34]. A combination between FBGs in passive fibers and double cladding designs for the active fibers was proposed and improved the slope efficiency (SE) up to 62% by using a single high reflectivity FBG (HRFBG), while an FBG pair (high reflectivity and low reflectivity), was ~5% lower [35]. On the other hand, previous completely monolithic designs, high and low FBG pairs inscribed into the active fiber, reported significantly lower SEs [36].

In this paper, results are presented for the development of a monolithic fiber laser operating in 1.95 μm using the plane-by-plane (pl-by-pl) femtosecond laser inscription method [37,38,39,40] directly into a thulium (Tm) fiber. A laser cavity was initially characterized with a single high reflectivity FBG in terms of effective length and power SE and then proceeded to develop three entirely monolithic cavities. All the lasers were characterized in terms of SE, effective length, and threshold power and the results were compared with previous work using the same Tm-doped fiber but having FBGs inscribed in silica passive fiber.

## 2. Materials and Methods

### 2.1. Fiber Bragg Grating Inscription and Characterization

The active fiber under test (FUT) used in this study was manufactured “in-house” by the Institute of Photonics and Electronics of the Czech Academy of Sciences (UFE). It is thulium-doped silica-based fiber (TDF) with core/cladding diameters of 6.7/125 μm and with a numerical aperture (NA) of 0.17. The attenuation loss was measured to be 0.02 dB/m, while the cutoff wavelength of the fiber was estimated to be close to 1500 nm. The core pumped TDF (4.5 mol % Al_2_O_3_, 2 mol % GeO_2_, and 0.4 mol % Tm^3+^) was fabricated using the solution doping method [41,42,43].

For the FBG inscription, a femtosecond laser (HighQ) operating at 517 nm with pulse duration 220 fs was used. The fiber sample was fixed on an air-bearing translation stage (Aerotech) with nanometer accuracy and the refractive index modifications were performed vertical to the core axis and limited only to the fiber core region. The laser repetition rate was set at 5 kHz and the pulse energy at ~100 nJ, as measured using a power meter at the output of the laser. The laser light was focused inside the core of the fiber without removing the outer protecting jacket, through a long working-distance lens and a mechanical stage.

**Figure 1:**
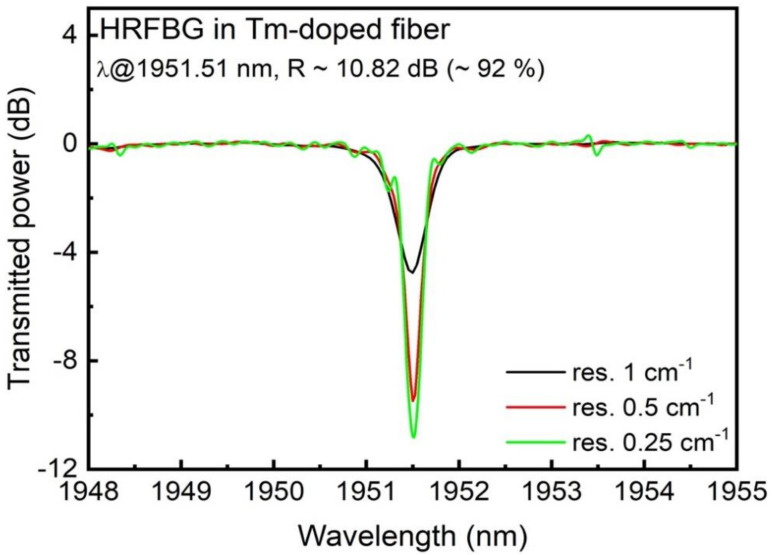
Transmission spectrum of a high reflectivity fiber Bragg grating (FBG) inscribed in highly doped thulium fiber using femtosecond laser inscription and measured in transmission, showing the importance of having sufficient measurement resolution.

Initial measurements were performed using a single HRFBG in order to estimate the effective length of the laser cavity. The HRFBG was a fourth order grating, inscribed with a period Λ = ~2.2 μm and 3000 periods, which resulted in a grating length of ~6.6 mm. The grating was characterized using an in-house build broadband light source [44] with an output power exceeding 100 mW and spectral range 1700–2050 nm. The output spectra were monitored with an FTIR spectrometer with a minimum spatial resolution of 0.25 cm^−1^, which is equivalent to ~325 pm. The resonance Bragg wavelength was found at 1951.5 nm, with an estimated strength exceeding 10 dB (~92% reflectivity) and a grating FWHM bandwidth of ~150 pm; the transmission spectrum is shown in Figure 1.

### 2.2. High Reflectivity Fiber Bragg Grating Characterization as Fiber Laser

**Figure 2:**
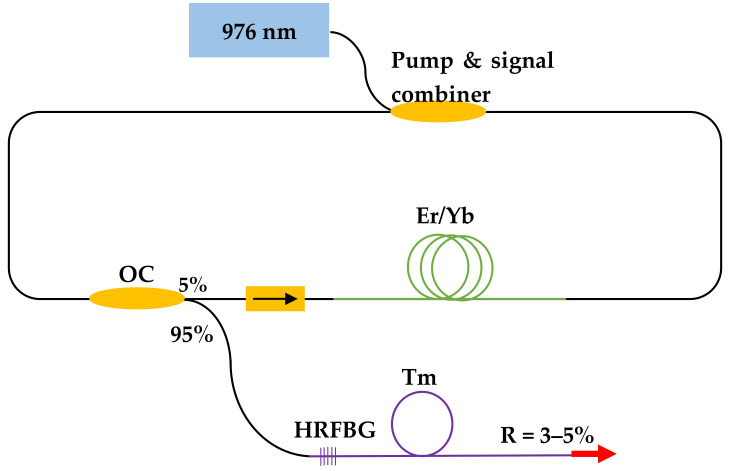
Schematic diagram of the experimental setup used to characterize the fiber laser (OC: optical coupler; Er/Yb: Erbium and Ytterbium doped fiber; Tm: Thulium doped fiber; HRFBG: High reflectivity FBG; R: reflection).

The HRFBG was inscribed at one end of a 4 m Tm-fiber length and the other end of the fiber was cleaved perpendicular to the fiber end-face to produce a low-reflectivity mirror (Fresnel reflection) and to demonstrate a Fabry–Perot cavity. An in-house-built erbium-doped fiber laser (EDFL) was fusion spliced <1 cm close to the HRFBG (Figure 2). The EDFL source had a maximum output power of 2.1 W at 1565 nm and the efficiency of the laser was measured for different fiber lengths using a digital power meter (Gentec). For these measurements, an optical filter (dichroic mirror DMLP 1800) with high reflection (HR) at 1560 nm and high transmission (HT) at 1.95 μm was placed in front of the power meter to separate the pump and signal beams; the optical spectra were recorded using a Miriad S3 spectrometer.

**Figure 3:**
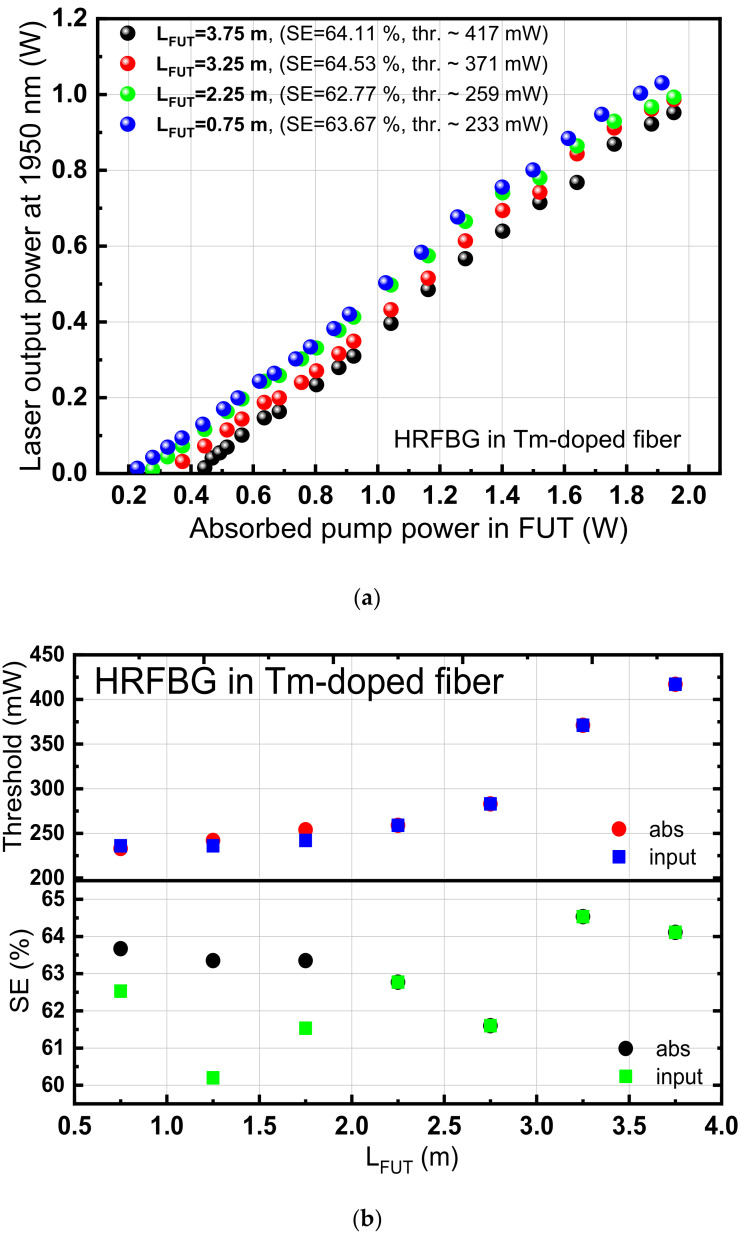
(**a**) Slope efficiency (SE) characterization results for different lengths (L_FUT_), for HRFBG, (**b**) threshold (upper) and SE in terms of length respective to the input and absorbed (abs) pump power at 1565 nm. (FUT: fiber under test).

**Figure 4:**
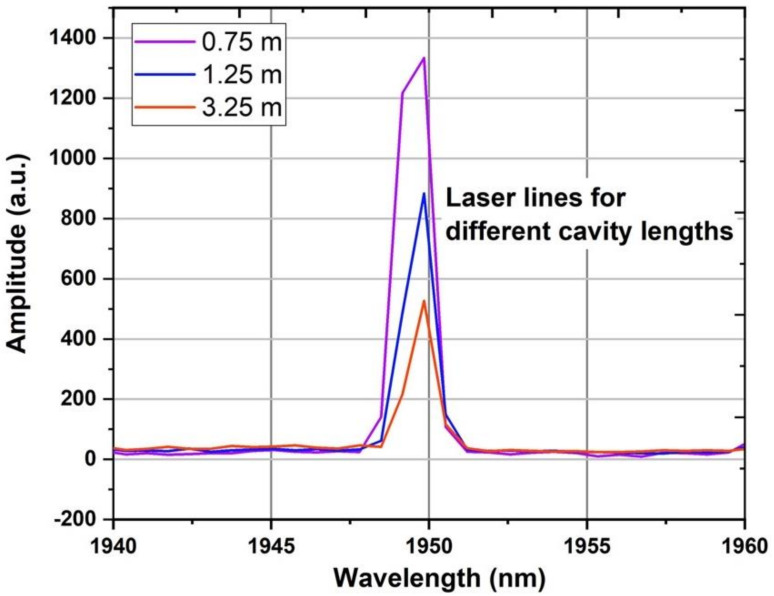
Optical spectra of the laser peaks for different cavity lengths, as recorded using the MIRIAD S3 spectrometer.

The FL was characterized in terms of effective length (starting with a length of active fiber ~4 m and gradually shortened to 0.75 m), threshold power, and power SE. The effective length of the fiber was found to be between 1 and 2 m. Regarding the SE (the dependence of output powers with respect to pump powers), it varied from 62.77 to 64.53%. The laser threshold was decreased as the fiber length was reduced (see Figure 3a,b), with the minimum laser threshold corresponding to 233 mW of pump power for an effective active fiber length of 75 cm. The SE and threshold power were calculated both with respect to the power of the diode source at 976 nm (absorption), which is the pumping source of the laser resonator respective to the output of the EDFL source (input), which acts as the input source pumping the active fiber. The SE is respective to absorbed power in active fiber (abs) and to the output of the EDFL source (input), which is the input pumping into the active fiber. The SE respective to absorbed power was found to be relatively flat for fiber lengths < 2 m. The laser peak positions of the FL output are presented in Figure 4 using the Miriad S3 spectrometer with a resolution ~1 nm. Given the limited spatial resolution of the spectrometer, no wavelength shift of the laser lines was noted since the laser wavelength is predefined only by the HRFBG.

### 2.3. Developoment of a Fully Monolithic Fiber Laser Cavity

Based on the preliminary results shown in the previous subsections, a fully monolithic fiber laser cavity was developed using an HR and low reflectivity FBG (LRFBG), replacing the end-face reflection from the perpendicular cleave. Using the same inscription parameters in terms of laser energy and repetition rate, three monolithic cavities inscribed were with similar length, in order to investigate the reproducibility of the inscription method. Specifically, the cavity lengths were 1.17, 1.16, and 1.09 m for the three cavities. As observed in the previous section, the SE of the lasers is almost flat for fiber lengths 0.75 m up to 2 m. As a result, centimeter-length differences between the samples are considered negligible with respect to the overall performance of the fiber laser. As before, all the cavities were characterized in terms of SE and threshold pump power.

**Figure 5:**
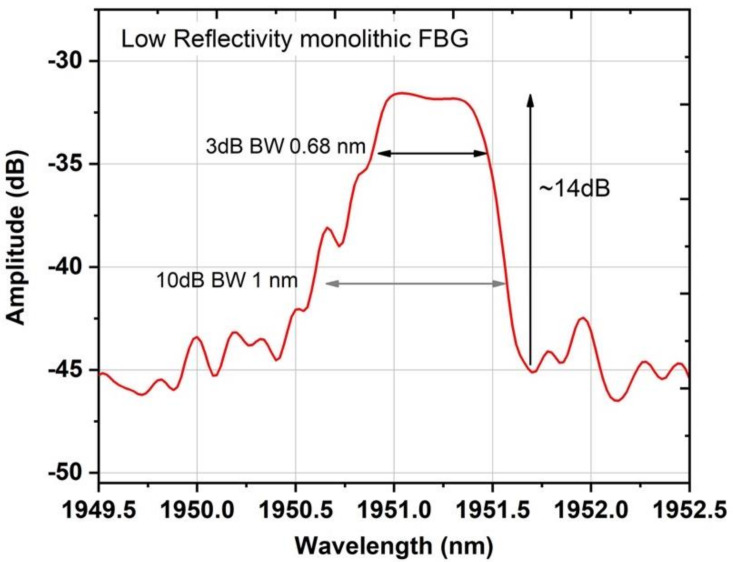
Reflection spectrum of the LRFBGs inscribed in Tm-doped silica fiber measured using a broadband light source and high-resolution optical spectrum analyzer.

During the inscription process using the plane-by-plane method, the refractive index change is constant for all of the grating planes. The grating strengthens and the bandwidth narrows with increasing periods (*N*) or grating length. Therefore, the FBG bandwidth (Δ*λ*_0_), is directly dependent on the FBG length, given the characteristic equation [45]:

(1) $$\frac{\Delta \lambda_{ο}}{\lambda_{Β}}=\frac{v\bar{\delta n}}{n_{eff}}\sqrt{1+{\left(\frac{\lambda_{Β}}{Lv\bar{\delta n}}\right)}^{2}}$$

where $\bar{\delta n}$ is refractive index change averaged over a grating period, ***v*** is the fringe visibility of the index change (1 is this case), ***n_eff_*** is the effective refractive index, and ***L*** is the length of the grating. Hence, fourth-order LRFBGs inscribed with N = 100 and total grating length ~200 μm resulted in a FWHM bandwidth of ~0.68 nm and 1 nm bandwidth at the 10 dB point (Figure 5). By controlling the bandwidth of the weak reflector (LRFBG), mismatching issues between the FBGs are avoided. However, due to the low reflectivity of the short-length LRFBGs, it was not possible to characterize their spectra in transmission with the available equipment but in reflection, as shown in Figure 5, using an optical circulator, an NKT Photonics (Birkerød, Denmark) supercontinuum source and a Yokogawa AQ6375B optical spectrum analyzer.

## 3. Results

**Figure 6:**
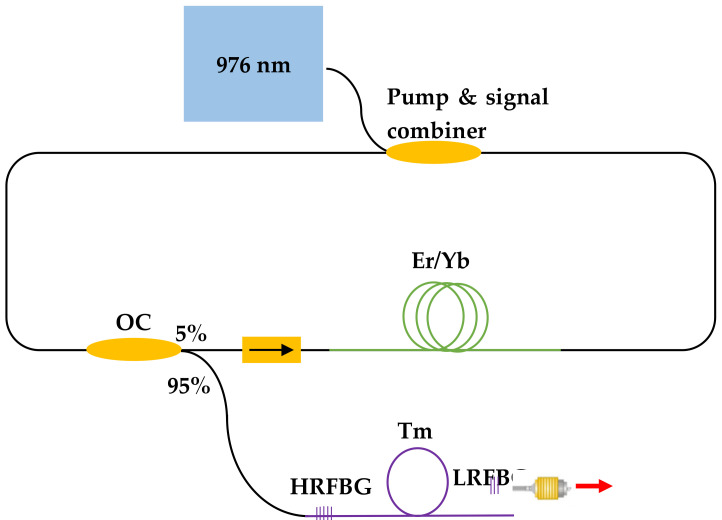
Experimental setup for characterizing the monolithic Fabry–Perot cavities inscribed in Tm-doped silica fiber.

**Figure 7:**
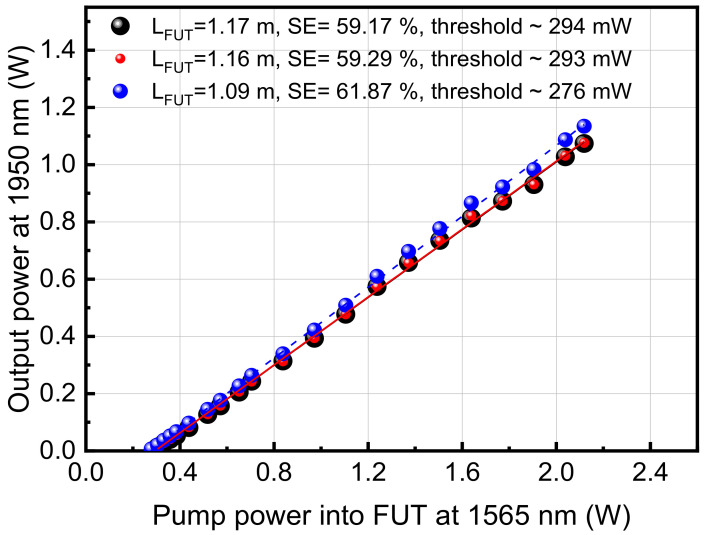
Slope efficiency of the three fiber lasers that were demonstrated using a monolithic pairs of FBG inscribed using the plane-by-plane femtosecond laser method.

**Figure 8:**
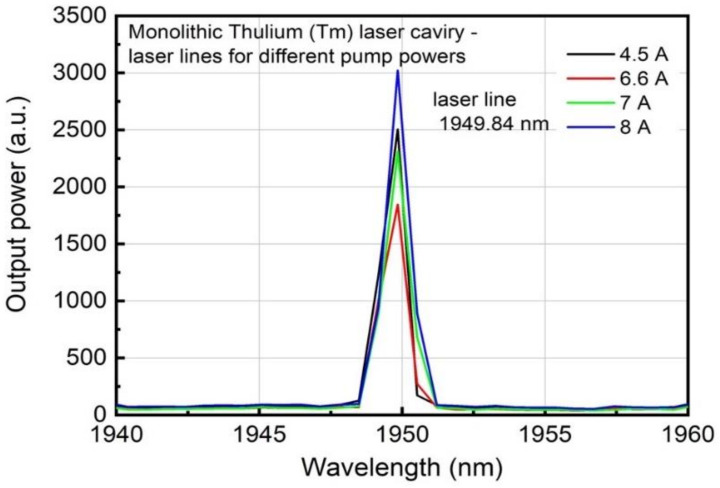

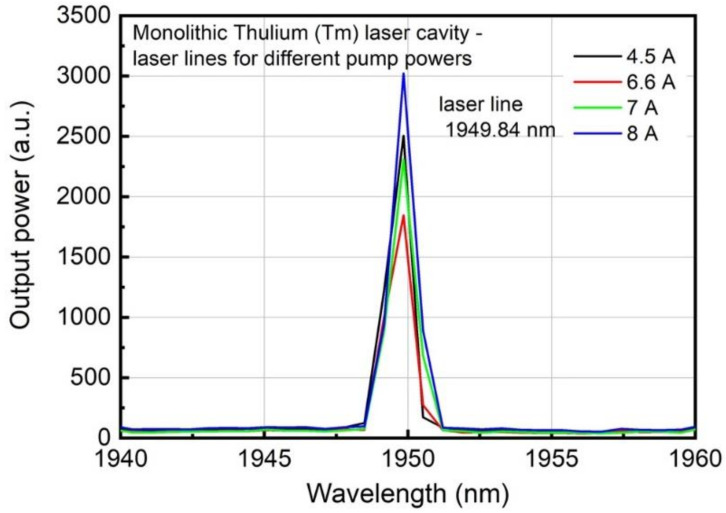
Laser lines of a monolithic Tm fiber laser developed using femtosecond laser inscribed FBGs directly into the active fiber for different pump powers.

The characterization results of the three monolithic fiber lasers, as performed using the configuration shown in Figure 6, are very similar (Figure 7). The SE varies from 59.17% to 59.29% for the FL strands with cavity length 1.17 and 1.16 m, respectively, and 61.87% for the shortest cavity length of 1.09 m. The threshold power was 294 mW for the 1.17 m cavity length and decreased to 276 mW for the 1.09 m cavity. Comparing the results with measurements using a single HRFBG, a small SE drop of ~4% was noted and an increase in threshold power, which is expected since the LRFBG induced some additional loss in the laser cavity and any remaining active fiber after the LRFBG absorbed light at 1950 nm. Typical laser spectra are shown in Figure 8, where a laser line at 1950 nm was determined by the FBGs. It is important to note that the output end was terminated with angle-polished (APC) pigtail in order to remove the back reflections from the end of the fiber and their contribution into the laser cavity [46,47]. There is no observable wavelength shift of the laser with increasing pump powers, within the measurement resolution limitations.

Table 1 summarizes for comparison the performance results of the monolithic fiber laser cavities presented in this work with the results presented in [8] using the same active fiber but commercial single HRFBG and FBG pair in passive fibers SMF28 (O/E Land, Inc). The monolithic design presented in this work significantly improved the SE compared to the passive FBG configuration. Specifically, an improvement >8% for the single HRFBG design is reported and more than 12% for the monolithic FBG pair compared to the fiber-spliced HRFBG and FBG pair configuration in passive fibers. On the other hand, the monolithic designs (HRFBG and FBG pair) have higher threshold power compared to the passive designs, because a portion of the pump is absorbed in doped parts of the fiber outside the laser resonator and this part of the pump does not contribute to the laser output.

## 4. Discussion

In this work, the highest slope efficiency was 61.87% respective to the input 1565 nm pumping power for a monolithic FBG pair (HRFBG + LRFBG), whereas for the single HRFBG design the slope efficiency reached 64%. Comparing the results of this work with previous studies using the same thulium-doped fiber [8], but using commercial FBGs in passive fibers, it was noted an improvement of >8% for the single HRFBG design and >13% for a fully monolithic design using HRFBGs and LRFBGs. There was a drop of ~2% on SE for the monolithic FBG pair when compared with the HRFBG design, which is related to absorption of the active fiber outside the laser cavity. In addition, for the same reason the threshold power was higher for the case of the monolithic pair compared with the HRFBG design, and the FBG pair in passive fibers.

The wavelength stability of fiber lasers developed using FBGs, depends upon two factors: (i) the fiber temperature, which increases as the pump power increases, and (ii) nonlinear effects, such as the population inversion of the Tm fiber. As reported in a previous study, a wavelength shift of 1.41 pm/mW was anticipated [36], as measured using a fiber with similar content of Tm and Al-dopant as utilized in this work, but for FBGs inscribed using a phase mask [8]. With respect to this value, the proposed monolithic FL cavities should have displayed a wavelength shift of >2.8 nm, since the lasers were pumped beyond 2 W. However, no such wavelength shift was observed, and indeed would be detectable using the available equipment (1 nm resolution). Hence, the anticipated wavelength shift was significantly lower than that reported in [36]. A possible reason could relate to the phase mask FBG inscription, where the whole area of the core and partially the cladding is modified, increasing absorption and loss.

In contrast, by using the pl-by-pl inscription method, the inscription laser pulse energy can be tailored to minimize losses resulting from the refractive index modifications, by precisely controlling the extent of the grating plane with respect to the size of the fiber core. It is highly important to have efficient femtosecond laser inscription methods for the development of monolithic fiber lasers, as it will be capable of inscribing gratings in other fiber types, such as fluoride fibers, expanding the existing technology to develop new and exciting applications. According to the current results, and in comparison with previous studies using the same fiber and passive FBGs, the femtosecond laser plane-by-plane inscription method can be used for the development of highly efficient monolithic fiber lasers (Table 1).

In addition, the spectral characteristics of the HRFBGs can be tailored according to the requirements of the applications. Particularly, the bandwidth of the grating can be tailored, resulting in a broader or thinner narrower laser line for finer high resolution gas sensing analysis.

## 5. Conclusions

In conclusion, a fully monolithic fiber laser was demonstrated, which could be applied to CO_2_ gas sensing in the short wavelength infrared region, using a single mode thulium-doped silica fiber as an active medium. The laser characteristics of a single high reflectivity FBG (>92%) inscribed directly into the Tm-doped in fiber laser configuration were measured and to experimentally identify the effective length of the fiber. Subsequently, three fully monolithic fiber laser cavities were demonstrated with similar inscription conditions in order to evaluate the reproducibility of the method. The cavities incorporated a short, broad bandwidth and low reflectivity FBG. The results show a high degree of reproducibility considering that all of the inscriptions were performed back-to-back without actively monitoring the gratings.

## Figures and Tables

**Table 1:** Summary of the Tm monolithic fiber lasers and comparison with laser configurations using FBGs in passive fiber.

Fiber Laser	SE (%)	Threshold (mW)	L_FUT_ (m)	Laser Line (nm)
HRFBG in SG1290 *	63.67	233	0.75	1950
Monolithic FBG pair *	61.87	276	1.09	1950
HRFBG in passive fiber [8]	55.14	180	1.17	1950
FBG pair in passive fiber [8]	48.62	214	3	1950

* This work.

## Data Availability

Not applicable.

## References

[1] Waynant R.W., Ilev I.K., Gannot I., Meyer J.R., Sirtori C. (2001). Mid-infrared laser applications in medicine and biology. Philos. Trans. R. Soc. A Math. Phys. Eng. Sci..

[2] Seddon A.B. (2013). Mid-infrared (IR)—A hot topic: The potential for using mid-IR light for non-invasive early detection of skin cancer in vivo. Phys. Status Solidi.

[3] Thomson M.A. (2014). Mid-IR Spectroscopy as a Tool for Cleanliness Validation.

[4] Zervas M.N., Codemard C.A. (2014). High Power Fiber Lasers: A Review. IEEE J. Sel. Top. Quantum Electron..

[5] Dragic P.D., Cavillon M., Ballato J. (2018). Materials for optical fiber lasers: A review. Appl. Phys. Rev..

[6] Theodosiou A., Aubrecht J., Peterka P., Kašík I., Todorov F., Moravec O., Honzátko P., Kalli K. (2018). Monolithic Er/Yb double-clad fibre laser with FBG inscribed using the direct-write plane-by-plane fs-laser inscription method. Proc. SPIE.

[7] Theodosiou A., Aubrecht J., Peterka P., Kasik I., Todorov F., Moravec O., Honzatko P., Kalli K. (2019). Er/Yb Double-Clad Fiber Laser With fs-Laser Inscribed Plane-by-Plane Chirped FBG Laser Mirrors. IEEE Photonics Technol. Lett..

[8] Aubrecht J., Peterka P., Honzátko P., Podrazký O., Kamrádek M., Proboštová J., Kašík I., Todotov F. (2017). Monolithic thulium-doped fiber laser. Photonics Devices Syst. VII.

[9] Voigtländer C., Thomas J., Wikszak E., Dannberg P., Nolte S., Tünnermann A. (2009). Chirped fiber Bragg gratings written with ultrashort pulses and a tunable phase mask. Opt. Lett..

[10] Hill K.O., Malo B., Bilodeau F., Johnson D.C., Albert J. (1993). Bragg gratings fabricated in monomode photosensitive optical fiber by UV exposure through a phase mask. Appl. Phys. Lett..

[11] Pospori A., Marques C.A.F., Sagias G., Lamela-Rivera H., Webb D.J. (2018). Novel thermal annealing methodology for permanent tuning polymer optical fiber Bragg gratings to longer wavelengths. Opt. Express.

[12] Marshall G.D., Williams R.J., Jovanovic N., Steel M.J., Withford M.J. (2010). Point-by-point written fiber-Bragg gratings and their application in complex grating designs. Opt. Express.

[13] Geernaert T., Kalli K., Koutsides C., Komodromos M., Nasilowski T., Urbanczyk W., Wojcik J., Berghmans F., Thienpont H. (2010). Point-by-point fiber Bragg grating inscription in free-standing step-index and photonic crystal fibers using near-IR femtosecond laser. Opt. Lett..

[14] Martinez A., Khrushchev I.Y., Bennion I. (2005). Thermal properties of fibre Bragg gratings inscribed point-by-point by infrared femtosecond laser. Electron. Lett..

[15] Jovanovic N., Fuerbach A., Marshall G.D., Withford M.J., Jackson S.D. (2007). Stable high-power continuous-wave Yb^3+-doped silica fiber laser utilizing a point-by-point inscribed fiber Bragg grating. Opt. Lett..

[16] Jovanovic N., Thomas J., Williams R.J., Steel M.J., Marshall G.D., Fuerbach A., Nolte S., Tünnermann A., Withford M.J. (2009). Polarization-dependent effects in point-by-point fiber Bragg gratings enable simple, linearly polarized fiber lasers. Opt. Express.

[17] Hudson D.D., Williams R.J., Withford M.J., Jackson S.D. (2013). Single frequency fiber laser operating at 2.9 µm. Advanced Solid-State Lasers Congress.

[18] Krämer R.G., Matzdorf C., Liem A., Bock V., Middents W., Goebel T.A., Heck M., Richter D., Schreiber T., Tünnermann A. (2019). Femtosecond written fiber Bragg gratings in ytterbium-doped fibers for fiber lasers in the kilowatt regime. Opt. Lett..

[19] Wikszak E., Thomas J., Burghoff J., Ortaç B., Limpert J., Nolte S., Fuchs U., Tünnermann A. (2006). Erbium fiber laser based on intracore femtosecond-written fiber Bragg grating. Opt. Lett..

[20] Wikszak E., Thomas J., Klingebiel S., Ortaç B., Limpert J., Nolte S., Tünnermann A. (2007). Linearly polarized ytterbium fiber laser based on intracore femtosecond-written fiber Bragg gratings. Opt. Lett..

[21] Peterka P., Kasik I., Dhar A., Dussardier B., Blanc W. (2011). Theoretical modeling of fiber laser at 810 nm based on thulium-doped silica fibers with enhanced ^3H_4 level lifetime. Opt. Express.

[22] Honzatko P., Baravets Y., Todorov F., Peterka P., Becker M. (2013). Coherently combined power of 20 W at 2000 nm from a pair of thulium-doped fiber lasers. Laser Phys. Lett..

[23] Todorov F., Aubrecht J., Peterka P., Schreiber O., Jasim A.A., Mrázek J., Podrazký O., Kamrádek M., Kanagaraj N., Grábner M. (2020). Active Optical Fibers and Components for Fiber Lasers Emitting in the 2-μm Spectral Range. Materials.

[24] Basov S., Milstein A., Sulimani E., Platkov M., Peretz E., Rattunde M., Wagner J., Netz U., Katzir A., Nisky I. (2018). Robot-assisted laser tissue soldering system. Biomed. Opt. Express.

[25] Fried N.M. (2018). Recent advances in infrared laser lithotripsy [Invited]. Biomed. Opt. Express.

[26] Böhm S., Schmidt M., Stichel T., Kahlmeyer M., Kryukov I., Sommer N. (2020). Single-step Laser Plastic Deposition (LPD) using a near-infrared Thulium fiber-laser. Polym. Test..

[27] Brosda M., Nguyen P., Olowinsky A., Gillner A. (2018). Laserwelding of biopolymers. Procedia Cirp.

[28] Mingareev I., Weirauch F., Olowinsky A., Shah L., Kadwani P., Richardson M. (2012). Welding of polymers using a 2μm thulium fiber laser. Opt. Laser Technol..

[29] Pal A., Chen S.Y., Sen R., Sun T., Grattan K.T.V. (2013). A high-Q low threshold thulium-doped silica microsphere laser in the 2 μm wavelength region designed for gas sensing applications. Laser Phys. Lett..

[30] Yao S., Chen S.Y., Pal A., Bremer K., Guan B.O., Sun T., Grattan K.T.V. (2015). Compact Tm-doped fibre laser pumped by a 1600 nm Er-doped fibre laser designed for environmental gas sensing. Sens. Actuators A Phys..

[31] Parriaux A., Hammani K., Millot G. (2019). Electro-optic dual-comb spectrometer in the thulium amplification band for gas sensing applications. Opt. Lett..

[32] Jackson S.D. (2004). Cross relaxation and energy transfer upconversion processes relevant to the functioning of 2 μm Tm3+-doped silica fibre lasers. Opt. Commun..

[33] Wu J., Yao Z., Zong J., Jiang S. (2007). Highly efficient high-power thulium-doped germanate glass fiber laser. Opt. Lett..

[34] Clarkson W.A., Pearson L., Zhang Z., Kim J.W., Shen D., Boyland A.J., Sahu J.K., Ibsen M. High Power Thulium Doped Fiber Lasers. Proceedings of the Optical Fiber Communication Conference and National Fiber Optic Engineers Conference.

[35] Tang Y., Huang C., Wang S., Li H., Xu J. (2012). High-power narrow-bandwidth thulium fiber laser with an all-fiber cavity. Opt. Express.

[36] Peterka P., Honzatko P., Becker M., Todorov F., Pisarik M., Podrazky O., Kasik I. (2013). Monolithic Tm-Doped Fiber Laser at 1951 nm With Deep-UV Femtosecond-Induced FBG Pair. IEEE Photonics Technol. Lett..

[37] Theodosiou A., Lacraz A., Polis M., Kalli K., Tsangari M., Stassis A., Komodromos M. (2016). Modified fs-Laser Inscribed FBG Array for Rapid Mode Shape Capture of Free-Free Vibrating Beams. IEEE Photonics Technol. Lett..

[38] Theodosiou A., Lacraz A., Stassis A., Koutsides C., Komodromos M., Kalli K. (2017). Plane-by-Plane Femtosecond Laser Inscription Method for Single-Peak Bragg Gratings in Multimode CYTOP Polymer Optical Fiber. J. Light. Technol..

[39] Fokine M., Theodosiou A., Song S., Hawkins T., Ballato J., Kalli K., Gibson U.J. (2017). Laser structuring, stress modification and Bragg grating inscription in silicon-core glass fibers. Opt. Mater. Express.

[40] Theodosiou A., Ioannou A., Kalli K. (2019). All-in-Fiber Cladding Interferometric and Bragg Grating Components Made via Plane-by-Plane Femtosecond Laser Inscription. J. Light. Technol..

[41] Cajzl J., Peterka P., Honzátko P., Podrazký O., Kamrádek M., Aubrecht J., Proboštová J., Kašík I. (2017). Evaluation of energy transfer coefficients in Tm-doped fibers for fiber lasers. Photonics Devices Syst. VII.

[42] Kamradek M., Honzatko P., Kasik I., Aubrecht J., Mrazek J., Podrazky O., Cajzl J., Varak P., Kubecek V., Peterka P. (2019). Nanoparticle and Solution Doping for Efficient Holmium Fiber Lasers. IEEE Photonics J..

[43] Kasik I., Peterka P., Mrazek J., Honzatko P. (2016). Silica Optical Fibers Doped with Nanoparticles for Fiber Lasers and Broadband Sources. Curr. Nanosci..

[44] Aubrecht J., Peterka P., Honzátko P., Moravec O., Kamrádek M., Kašík I. (2020). Broadband thulium-doped fiber ASE source. Opt. Lett..

[45] Erdogan T. (1997). Fiber grating spectra. J. Light. Technol..

[46] Peterka P., Navrátil P., Maria J., Dussardier B., Slavík R., Honzátko P., Kubeček V. (2012). Self-induced laser line sweeping in double-clad Yb-doped fiber-ring lasers. Laser Phys. Lett..

[47] Peterka P., Koska P., Ctyroky J. (2018). Reflectivity of Superimposed Bragg Gratings Induced by Longitudinal Mode Instabilities in Fiber Lasers. IEEE J. Sel. Top. Quantum Electron..

